# Correlation of Fall Height, Fracture Severity and Clinical Outcome in Pediatric Supracondylar Fractures—A Retrospective Analysis with an Observation Period of 20 Years

**DOI:** 10.3390/children10030510

**Published:** 2023-03-04

**Authors:** Andrea Schuller, Sebastian Hahn, Lorenz Pichler, Anna Hohensteiner, Thomas Sator, Manuela Jaindl, Elisabeth Schwendenwein, Thomas Tiefenboeck, Stephan Payr

**Affiliations:** Department of Trauma Surgery, University Clinic of Orthopedics and Trauma Surgery, Medical University of Vienna, 1090 Vienna, Austria

**Keywords:** pediatric injuries, supracondylar humerus fractures, injury mechanism, fall height, clinical outcome, correlation

## Abstract

The most common cause leading to supracondylar humerus fractures in children is falling onto an outstretched arm. A correlation between fall height and fracture severity may be assumed but has not yet been described. The aim of this study was to show that fracture severity increases with fall height. Furthermore, the correlation between fracture severity and outcome was examined. A total of 971 children with supracondylar humerus fractures between January 2000 and December 2019 were included in this study. The correlations between fall height and fracture severity and between fracture severity and outcome were assessed. Increasing fall height correlates with fracture severity (*p* < 0.001; r = 0.24). Furthermore, the incidence of complications increases with fracture severity and a correlation was present accordingly (*p* < 0.001; r = 0.28). A total of 30 (3.1%) patients showed limitations in range of motion and/or persistent neurologic deficits at the latest follow-up. Type I fractures rarely lead to subsequent limitations. The correlation between increasing fall height and fracture severity was significant. Furthermore, children with type III and IV supracondylar fractures are more likely to develop complications or restrictions in movement than children with type I and II fractures. Hence, the initial fall height may be an indirect indicator of a more or less favorable outcome.

## 1. Introduction

Supracondylar humerus fractures are one of the most common injuries in childhood and adolescence with an incidence of 14% [1]. In the elbow joint, supracondylar humerus fractures account for more than two-thirds of all injuries [2,3] and occur most frequently between the age of 5 and 7 years [4,5,6]. The gender distribution of patients with supracondylar humerus fractures as well as the eventual differences in injury mechanism, pattern, therapy and outcome are controversially discussed in the literature [5,7,8]. The most common cause of injury leading to supracondylar humerus fractures is children falling from various heights onto an outstretched arm [9]. These fractures occur in both low-energy trauma, such as playground accidents, and high-energy trauma, such as sports [9]. Supracondylar humerus fractures are commonly classified according to the Von Laer classification, which distinguishes four fracture types of increasing severity [10,11,12].

Since type I fractures do not involve joint dislocation and are considered stable, these fractures are treated conservatively by a sufficient immobilization with upper-arm casts or Blount slings [13]. The immobilization can be terminated after two to three weeks and movement can be started. Because of the benign character of the type I fracture and the generally conservative treatment, the rate of complications is low and the outcome is likely preferable [14]. Minor growth disturbances because of unevenly rapid growth may be mentioned as complications. Type II fractures are at risk of dislocation. The difficulty with type II fractures is to distinguish from a radiograph whether there is a total dislocation through the trochlea or an incomplete fracture line in which the cartilaginous trochlea is still intact. These fractures must be evaluated after 4 days because secondary dislocation may occur, requiring surgical treatment [15]. The adequate evaluation of the stability of Von Laer type II fractures is essential for their treatment. In the case of instability, conservative treatment should be avoided and surgical treatment must be considered [15]. As with all surgeries, there is a certain risk of suffering surgery-associated complications. Possible rotational malalignment and inadequate reduction in the proximal fracture element may result in growth disturbances and the formation of a cubitus varus/valgus. Inadequate diagnosis in primary care is most likely responsible for the occurrence of cubitus varus, and already existing fracture deformities can be overlooked. However, these complications currently occur less frequently [15]. During surgical treatment, ulnar nerve injury can occur with an incidence of up to 15% when the Kirschner-wire (K-wire) is inserted [10,16,17]. In type III and IV fractures, the distal fragment is dislocated in two or three planes. Instability with complete dislocation of the fracture element and risk of nerve and vascular injury is an absolute indication for surgery [10,11,12,14,15,18]. Accordingly, type III and IV fractures are at risk of developing growth disturbances, nerve-vascular damage and deformities (cubitus varus or valgus) [15,17]. Conservative treatment usually consists of the application of an upper-arm cast in a 60–100° flexion position for about two to three weeks if possible. The lower arm is fixed in a neutral position [14,19]. Surgical treatment of supracondylar humerus fractures is usually performed by K-wire osteosynthesis. Countless studies have been conducted to investigate the configuration of the K-wires in order to offer an optimal treatment [20,21,22,23,24]. In the case of surgical treatment of unstable fractures, closed reduction and subsequent fixation with K-wires is usually performed. If closed reduction is not possible, open reduction might be necessary. Other reasons for performing open reduction may be the treatment of an open wound, compartment syndrome or neurological and vascular lesions [25,26]. Pinning techniques are available in different arrangements. At our department, the crossed bilateral variant with two K-wires is used with each wire crossing the other, ascending from distal radial and ulnar to proximal. On the one hand, the ulnar nerve is endangered while inserting the K-wire from the medial, and on the other hand, this bilateral variant is one of the most stable forms of fixation using K-wires [21,22,27]. Generally, this method is described as effective and safe in the literature [28]. Further methods that have been described are elastic stable intramedullary nails (ESIN), plating/screwing and applying an external fixator [15]. Since falling on an outstretched arm is the most common mechanism of injury [9], a correlation between the height of the fall and the severity of the fracture may be assumed. However, there are currently no studies to describe this. The aim of this study was to show that fracture severity increases with fall height. In addition, the correlation between fracture severity and clinical outcome was examined.

## 2. Materials and Methods

**Figure 1:**
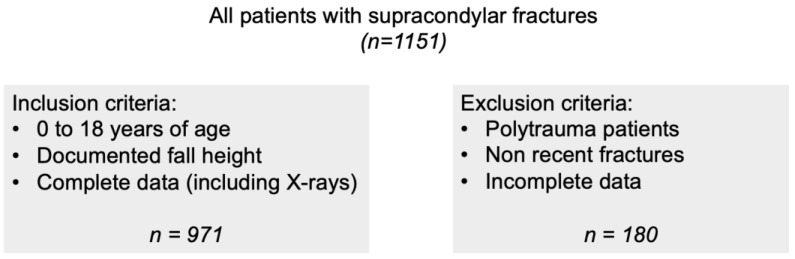
Inclusion and exclusion criteria.

This retrospective study was conducted with the approval of the Ethics Committee of the Medical University of Vienna (Code 1839/2020) and according to the Declaration of Helsinki in its latest amendment. In total, 1151 children and adolescents aged from 0 to 18 years were treated with supracondylar humerus fractures at the University Clinic of Trauma Surgery at the Medical University of Vienna, during an observation period from January 2000 to December 2019. After applying the inclusion and exclusion criteria, 971 children with radiological evidence of a supracondylar humerus fracture were included (Figure 1).

**Figure 2:**
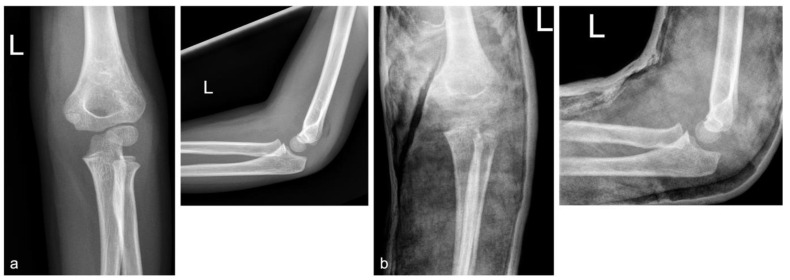
The course of a conservatively treated type II supracondylar fracture of the left (L) arm of a 5-year-old girl who fell from a standing position. (**a**) Initial anterior–posterior (a.p.) and lateral X-rays showing a type II fracture according to the Von Laer classification. (**b**) A.p. and lateral X-rays within the upper-arm cast (for three weeks) showing the fracture in an unchanged position (approximately 4 days after injury for exclusion of a secondary dislocation). Patient presented full range of motion and no deficits two weeks after cast removal.

**Figure 3:**
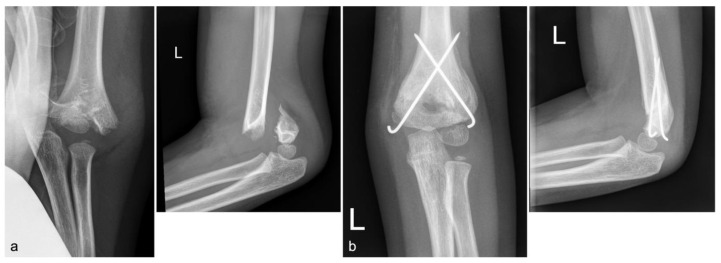
The course of a surgically treated type IV supracondylar fracture of the left (L) arm of a 6-year-old boy who fell from a climbing frame (1.5 m). (**a**) Initial a.p. and lateral X-rays showing the type IV fracture according to the Von Laer classification. The boy was operated on the same day. (**b**) A.p. and lateral X-rays after bilateral K-wire fixation 6 weeks postoperatively with new bone formation. Implant removal was performed 7 weeks after the initial surgery. The patient had no deficits 4 weeks after implant removal.

Data were collected retrospectively from patient charts, including age, sex, injury mechanism and treatment (surgical or conservative). Regarding the injury mechanism, an additional survey of the height of the fall was carried out. Patients were divided into the following categories: “fall from standing position”, “fall height below 1 m” and “fall height over 1 m”. The fall height and the injury mechanism were documented in every reported case (inclusion criteria). These data are demanded by our standards and medical report. Parents are always asked how high the fall was. Therefore, a fall height is documented in every medical report. Falls from a height below 1 m were usually falls from a bench or a couch. Falls from more than 1 m were usually injuries in parks from a climbing frame. However, in all cases parents were asked how high the couch, bench, diaper changing table, climbing frame, etc., was; therefore, the fall height and the differentiation between below or over 1 m was documented through the assessment of the parents who witnessed the injury. Fractures were classified using the Von Laer classification (type I-IV) [12]. Conservative treatment consisted of the application of an upper-arm cast in a 60–100° flexion position for about two to three weeks if possible (Figure 2). The lower arm was fixed in a neutral position. X-rays were performed after one week and after the removal of the cast. Movement was then allowed and patients were rescheduled for a further appointment after two weeks. The surgical treatment and rehabilitation protocol foresaw the bilateral K-wire fixation technique (Figure 3). A postoperative wound check was performed on the second day. Removal of stitches, applying a new cast and X-ray control were performed after 12–14 days. The cast was removed after three weeks, and then full range of movement was allowed as for the conservatively treated patients. The next appointment was scheduled for two weeks later. Further appointments were scheduled according to the clinical presentation and subjective patient satisfaction. Implant removal was usually planned for six weeks after the initial surgery after obtaining one prior X-ray. Complications such as growth disturbances, limitations in the range of motion (ROM) and neurological deficits were recorded at the latest follow-up to document the clinical outcome.

### Statistical Analysis

Descriptive data were reported for the entire patient cohort including mean ± standard deviation. Nominal and ordinal variables were presented as absolute and relative frequencies. Metric variables were reported as mean, range and standard deviation. The confidence interval for relative frequencies was 95%. In order to assess a correlation between the height of fall and the severity of the fracture, a Spearman test was used. The outcome was described with the following parameters: “limitation in the range of motion at the last FUP (yes/no)”, “radiologically or clinically relevant growth disturbances (yes/no)” and “complications after treatment (yes/no)”. For all of these parameters combined, a biserial rank correlation between the classification and outcome was performed. The significance level was set at a *p*-value of <0.05. The Bonferroni–Holm correction method was applied to account for multiple comparisons.

Statistical analysis was performed using Microsoft Excel (Version 16.50.; Microsoft Corp., Redmond, WA, USA) and GraphPad Prism (Version 9.4.1 for Mac; GraphPad Software, San Diego, CA, USA).

## 3. Results

### 3.1. Demographic Data

The entire patient cohort involved 971 children with a mean age of 5.2 ± 2.8 years (male: 523; mean age: 5.5 ± 2.9 years; female: 448; mean age: 4.9 ± 2.6 years) within an observation period between January 2000 and up to and including December 2019. During this duration, an overall increase in supracondylar humerus fractures was documented (Table 1). When comparing the two decades, a simultaneous increase in supracondylar humerus fracture by 7% occurred in female and male patients.

Generally, the most common injury mechanism was falling on an outstretched arm, specifically, falls from a standing position (510; 52.6%), including falls while walking or running. With a fall height below 1 m (241; 24.8%), children fell from sofas, beds and jumped from or over various objects. Incidents with a fall height of more than 1 m (219; 22.6%) occurred in sports or at the playground and in everyday life. With 544 fractures (56.1%), type I fractures accounted for the largest proportion of patients observed. Type II to IV fractures occurred with approximately equal frequency, with type III fractures accounting for the smallest proportion (107; 11.0%) of all fractures. The gender distribution according to the four fracture types is listed in Table 2. 

A total of 623 (64.2%) patients were treated conservatively by immobilization with an upper-arm cast, while the remaining 348 (35.8%) patients were treated surgically by bilateral K-wire fixation. A more detailed presentation of the treatment according to the fracture type is listed in Table 3, and the frequency of an open or closed reduction is presented in Table 4. The overall mean follow-up was 66 ± 130.9 days for the entire cohort. The mean follow-up for the conservatively treated patients was 31.7 ± 53.9 and for the surgically treated patients 123 ± 189.9 days.

### 3.2. Correlation of Fall Height and Type of Fracture

Descriptively, falls from a standing position generally resulted in type I fractures (330/971; 34.0%) (Table 5). An increasing fall height seems to lead to increased severity of fractures according to the fracture classification. Type IV fractures predominantly occurred after falls from heights of more than 1 m (61/971; 6.3%). By using the Spearman test, a correlation between the fall height and the classification was shown (*p* < 0.001; r = 0.24). 

### 3.3. Outcome

#### 3.3.1. Complications after Treatment

A total of 52 (5.4%) patients experienced 53 complications. Complications after conservative treatment included secondary dislocation (4; 0.4%). The majority of complications occurred after surgery (48; 4.9%) and included 21 nerve lesions, 18 K-wire-induced skin lesions, 2 vascular injuries, 4 postoperative infections and 6 dislocations of a fracture fragment. Six of these fifty-two patients received a surgical revision, including neurolysis of the ulnar nerve when the implant removal was performed in four patients and because of secondary dislocations in the remaining two patients. The incidence of complications increased with the severity of the fracture with most complications occurring in type IV fractures (18; 1.9%). By trend, complications after treatment seem to be more likely to occur in male patients (Table 6). 

#### 3.3.2. Limitations of Range of Motion (ROM)/Neurological Deficits

A total of 30 (3.1%) patients showed limitations of ROM or persistent neurologic deficits at the latest follow-up. A total of 19 of these patients reported no clinical restrictions in daily life despite minimal limitations in ROM. In the conservative group, a total of four patients (0.4%) were observed with flexion deficits. In the surgical group, 25 (2.7%) manifested flexion deficits (10; mean 13.0 ± 8.2 degrees) and extension deficits (11; mean 9 ± 8.0 degrees) and 4 neurological deficits of the ulnar nerve. Three patients with ulnar nerve lesions reported both sensory and motoric deficits but were still improving at the latest follow-up. As type I fractures rarely lead to subsequent limitations, only one patient with a type I fracture presented a flexion deficit of 10 degrees. However, 8 patients with type II fractures and 10 patients with type III and IV fractures each showed limitations of mobility and/or neurological deficits at the latest follow-up. These adverse events do not reveal any relevant differences concerning the gender distribution. 

#### 3.3.3. Growth Disturbances

Nine (0.9%) patients developed growth disturbances including varus and valgus deformities. The valgus deformities (5; 0.5%) ranged from 5 to 14 degrees with a mean of 7.2 ± 3.9 degrees, and the varus deformities (4; 0.4%) from 5 to 10 degrees (mean 7.5 ± 2.9). All nine deformities were only radiologically noticeable and had no effects on the patient’s daily life and were therefore clinically irrelevant. Despite the fact that these observed growth disturbances were clinically irrelevant, they were strictly considered potential adverse events and included as complications in the correlation. Of these nine patients, three with type I fractures developed deformities after conservative treatment, and the remaining six patients with more severe fractures (type II, III, IV) were treated surgically. By trend, growth disturbances seem to occur more often in male patients. The adverse events according to the fracture classification are presented with the gender distribution in Table 6.

#### 3.3.4. Correlation of Type of Fracture and Outcome

Descriptively, 83 children developed a total of 91 adverse events that were associated with a complication after treatment, a limitation of ROM/neurological deficit or a radiologically or clinically relevant growth disturbance (Table 7). The increased fracture severity linked to the fracture classification correlated with an increased risk of developing an adverse event (*p* < 0.001; r = 0.28). 

## 4. Discussion

The increasing frequency of pediatric supracondylar humerus fractures in recent years, which is reflected in the results of this work, has also been described by other authors, such as LiBrizzi et al. and Barr et al. [7,29]. Although a large amount of existing literature on the issue of pediatric supracondylar humerus fractures exists, only a few comparable and representative epidemiological series of data are available [3,10,13,30,31,32,33]. The long observation period of 20 years and the large study population clearly stands out from previous comparable studies with lower study populations and observation periods with a maximum of five years. LiBrizzi et al. published the only paper so far in 2020, which is a single-center study with a comparable number of patients as in the current manuscript [7]. However, the study by LiBrizzi et al. was conducted at John Hopkins Hospital in the USA, primarily focused on health-sex differences and did not publish data on follow-up and outcome. Comparability is limited due to a different healthcare system and different treatment standards. With reference to the patient cohort of this study of in total 971 patients, the female: male ratio was 1:1.2, as more male (523; 53.9%) than female (448; 46.1%) patients were treated for a supracondylar humerus fracture. This gender difference is also shown in previous studies that depicted a higher frequency of male patients by about 60% [3,5,6,8,18. Apart from that, other studies describe an equal incidence [19,34] or even a female predominance as shown by LiBrizzi et al. who referred to further studies showing this trend [35,36]. Although there are some studies showing a higher incidence of female patients, the majority of cases are predominated by boys assumed to engage in increased risk behavior or “rougher” play, but this has only been suspected [7]. Furthermore, we illustrated an increase in supracondylar humerus fractures both in male and female patients similar to the findings of LiBrizzi et al. In terms of fracture types, a related ratio of female to male patients was reflected in all fracture types (1:1.2) with the highest difference in type II fractures (1:1.4), which is comparable to other studies as LiBrizzi et al. stated no significant gender differences in fracture characteristics. As the present study displayed no gender differences in fracture types, there was the same gender distribution in conservative and surgical treatment with a slight majority of male patients, comparable to the findings of LiBrizzi et al. While the gender distribution of the frequency of patients with complications or limitations of the ROM was consistent with the gender distribution of the study population as a whole, more than twice as many male patients developed growth disturbances after treatment, but these were clinically irrelevant. Therefore, in general, there were no differences in the frequency of complications, similar to the findings of LiBrizzi et al. Studies have discussed that the force on the elbow during a fall may influence the extent of nerve, tissue and vascular lesions and operation time [31,34,37,38]. However, a correlation between fall height and the extent of dislocation has not been presented in any study to date. Thus, this present study is the first to point out a significant correlation between fall height and fracture severity according to the Von Laer classification (r = 0.24; *p* < 0.001). Descriptively, it also illustrates that type I and II fractures arose at a lower fall height, while type III and IV fractures predominately occurred per percentage with increasing height. Furthermore, it could be shown that the complication rate significantly correlated with increased fracture severity. Such an association is also suggested by Mitchelson et al. [32] and may be connected to the fact that severe fractures often require surgical treatment which increases the risk of perioperative complications [39]. Reflecting this, in our study population, the majority of complications (48/52) occurred in surgically treated patients that also mostly suffered type III or IV fractures (35/52). Data also show that in only half of the patients who obtained a type IV fracture could a closed reduction be performed. The majority of type IV fractures made an open reduction necessary to finally perform a bilateral crossed K-wire fixation. Given the presented complication rate and the fact that bilateral incisions (unfavorable cosmetic aspects) are sometimes necessary, the question could arise whether other methods (e.g., external fixator) may reduce this risk. In this study, all children who received surgery were operated on in the supine position. In the literature, no differences have been described in terms of functional and radiological outcomes between prone and supine positioning, and positioning is dependent on the habits and experience of the surgeons and anesthesiologists [40]. In addition, this study showed that although the majority of patients were clinically fully functional at the end of their treatment, functional and neurological deficits scarcely occurred in type I fractures. Most of the deficits occurred in patients with type III and IV fractures. Type III and IV fractures led to a higher number of complications and deficits than type I and II fractures, prognosticating an impaired outcome. Since the severity of the fracture increased with the fall height, this may also be a predictor of the outcome of an obtained supracondylar fracture. The limitation of this study is the retrospective design. On the one hand, this design allows for a very precise analysis of exact data, such as age, gender, radiographs and time periods, but on the other hand, it may lead to possible bias since examination and documentation differs depending on the physician. The patient-related data were conducted at a level 1 trauma center. However, the department represents the largest trauma surgery department, including pediatric trauma, in the country; therefore, the data of this monocentric study can be considered representative.

## 5. Conclusions

This study has shown a significant correlation between increasing fall height and fracture severity in supracondylar humerus fractures classified according to Von Laer. Furthermore, children with type III and IV supracondylar fractures are more likely to develop complications or restrictions in movement than children with type I and II fractures. Data illustrate that the more severe the fractures (type III and IV) are, the higher the risk of experiencing complications and an impaired outcome. Hence, the fall height may be an indirect indicator of a more or less favorable outcome. 

## Figures and Tables

**Table 1:** Time course of the frequencies of infantile supracondylar humerus fractures in girls and boys.

	2000–2009	%	2010–2019	%	Total	%
**Female**	189	19.5	259	26.7	448	46.1
**Male**	223	23.0	300	30.9	523	53.9
**Total**	412	42.4	559	57.6	971	100.0

**Table 2:** Gender distribution according to the four fracture types classified by Von Laer.

	Female	%	Male	%	Total	%
**Type I**	258	26.6	286	29.5	544	56.0
**Type II**	72	7.4	102	10.5	174	17.9
**Type III**	52	5.4	56	5.8	108	11.1
**Type IV**	66	6.8	79	8.1	145	14.9
**Total**	448	46.1	523	53.9	971	100.0

**Table 3:** Treatment modality according to fracture types.

	Conservative	%	Operative	%	Total	%
**Type I**	531	54.7	13	1.3	544	56.0
**Type II**	89	9.2	85	8.8	174	17.9
**Type III**	3	0.3	105	10.8	108	11.1
**Type IV**	0	0.0	145	14.9	145	14.9
**Total**	623	64.2	348	35.8	971	100.0

**Table 4:** Frequency of open and closed reduction according to fracture types.

	Open	%	Closed	%	Total	%
**Type I**	6	1.7	7	2.0	13	3.7
**Type II**	15	4.3	70	20.1	85	24.4
**Type III**	35	10.1	72	20.7	105	30.2
**Type IV**	91	26.1	54	15.5	145	41.7
**Total**	147	42.2	203	58.3	348	100.0

**Table 5:** Fracture classification according to fall height.

	Fall from Standing Position	%	Fall from <1 m	%	Fall from >1 m	%	Total	%
**Type I**	330	34.0	136	14.0	78	8.0	544	56.0
**Type II**	78	8.0	48	4.9	48	4.9	174	17.9
**Type III**	54	5.6	22	2.3	32	3.3	108	11.1
**Type IV**	49	5.0	35	3.6	61	6.3	145	14.9
**Total**	511	52.6	241	24.8	219	22.6	971	100.0

**Table 6:** Gender distribution concerning adverse events and fracture classification.

	Complications after Treatment				Limitation of ROM/ Neurological Deficits				Growth Disturbances			
	Female	Male	Total	%	Female	Male	Total	%	Female	Male	Total	%
**Type I**	1	4	5	9.6	3	0	3	10.0	1	2	3	33.3
**Type II**	6	7	13	25.0	4	4	8	26.7	0	1	1	11.1
**Type III**	5	11	16	30.8	3	6	9	30.0	0	4	4	44.4
**Type IV**	7	11	18	34.6	4	6	10	33.3	1	0	1	11.1
**Total**	19	33	52	100.0	14	16	30	100.0	2	7	9	100.0

**Table 7:** Adverse events according to fracture classification.

	Complications after Treatment	%	Limitation of ROM/Neurological Deficits	%	Growth Disturbances	%
**Type I**	5	0.5	3	0.3	3	0.3
**Type II**	13	1.3	8	0.8	1	0.1
**Type III**	16	1.6	9	0.9	4	0.4
**Type IV**	18	1.9	10	1.0	1	0.1
**Total**	52	5.4	30	3.1	9	0.9

## Data Availability

The datasets generated and/or analyzed in the current study are not publicly available due to data privacy but are available from the corresponding author on reasonable request.
